# Effects of the ECHO tele-mentoring program on Long COVID management in health facilities in India: A mixed-methods evaluation

**DOI:** 10.1371/journal.pone.0331293

**Published:** 2025-11-11

**Authors:** Rajmohan Panda, Ritika Mukherjee, Kalpana Singh, Supriya Lahoti, Apoorva Karan Rai, Amit Verma, Sandeep Bhalla, Haresh Chandwani, Kumud Rai, Archisman Mohapatra

**Affiliations:** 1 Independent Consultant, ECHO India, New Delhi, India; 2 Generating Research Insights for Development (GRID) Council, Noida, Uttar Pradesh, India; 3 Consultant Biostatistics, ECHO India, New Delhi, India; 4 Extension for Community Healthcare Outcomes (ECHO) India, New Delhi, India; Maulana Azad Medical College, INDIA

## Abstract

**Background:**

Long COVID emerged as a significant long-term consequence of COVID-19 characterized by persistent symptoms post-infection. ECHO India initiated a training program across four states to enhance the capacity of medical officers (MOs) to manage long COVID syndrome. This study was undertaken to evaluate the effect of the ECHO tele-mentoring program on long COVID management in public health facilities in terms of change in knowledge, competence, and performance of the trained MOs.

**Methods:**

Mixed-methods approaches were adopted. Moore’s Expanded Outcomes Framework was used for the study. Differences between the pre- and post-interventions were used to populate levels 1–5 of the framework with the trained MOs. This was supplemented by key informant interviews with stakeholders, i.e., trained MOs, hub leaders, and trainers. Level 6 was evaluated with patients seeking services for long COVID from the trained MOs. through quantitative exit interviews and in-depth interviews in two intervention states.

**Results:**

The pre-post analyses were conducted on a sample size of 204 MOs; a total of 420 beneficiary patients were surveyed. In-depth interviews were done with another 20 patients to measure satisfaction. The findings reveal a significant increase in the MOs’ knowledge, learning, and competence. MOs expressed appreciation for the interactive nature of the tele-mentoring sessions and reported increased confidence in dealing with long COVID cases. The training improved the MOs’ focus on mental health as a treatment strategy for long COVID. Patients interviewed expressed satisfaction with the care provided by the MOs, in particular with communication skills and the comprehensive approach adopted for long COVID management. They valued the information, the thorough examinations, and the recommendations given by the trained MOs.

**Conclusion:**

The ECHO tele-mentoring program improved the knowledge and skills of primary care medical officers and also resulted in patient satisfaction.

## Introduction

“Long COVID” is a multisystem disease that lasts for four or more weeks following initial symptoms of COVID-19 (with or without a positive test) [1]. It includes both ongoing symptomatic COVID-19 (4–12 weeks) and post-COVID-19 syndrome (signs and symptoms that continue for more than 12 weeks) [1,2]. Studies in India have estimated an overall incidence of Long COVID from 22% to 30% [3–5]. In severe to critical COVID-19 cases, the estimated incidence of long COVID ranges from 62 to 74% [3]. In low- and middle-income countries (LMICs), long COVID may have a severe impact due to existing limited healthcare resources, inadequate healthcare infrastructure, poor service awareness and access, and higher pre-existing conditions [6]. The functional disability associated with long COVID can hinder an individual’s capacity to perform daily tasks and fulfil employment responsibilities, leading to socioeconomic consequences [5].

The need for clinicians to receive evidence-based information on long COVID management has been well documented. The gap in the provision of such information can be addressed through regular training [7–9]. Good training can yield dividends in terms of improved work performance, quality of care, patient satisfaction, and thus, leading to a healthier community [10–13]. Project Extension for Community Healthcare Outcomes (ECHO) in collaboration with the State health departments (State National Health Mission (NHM) facilitated the training of 1000 Medical Officers (MOs) for the management of long COVID syndrome through the online ECHO tele-mentoring model across 4 states in 2022−23.

We evaluated the effects of the above-mentioned ECHO tele-mentoring program in public health facilities using a mixed-methods approach. We sought to answer two key questions: a) to what extent did the training change the knowledge, competence and performance of the MOs for long COVID management? and b) what were the patient experiences (satisfaction) with those services and their recommendations for improving service delivery for their condition? This paper presents details of the study.

## Materials and methods

### Study (evaluation) design

We used a single-group (self-controlled) pre-post quasi-experimental design. A quasi-experimental design was chosen since randomizing the medical officers or the selection of matched controls for the training was not feasible. We used mixed-methods approaches. Integration of the quantitative and qualitative components was achieved at the time of data analysis and interpretation.

### Framework of evaluation and approach

We used Moore’s Expanded Outcomes Framework for Assessing Learners and Evaluating Instructional Activities [14,15]. The study included two work packages (Supplementary File 1). Workpackage #1 (WP#1) assessed provider-side effect using a pre-post design with the same set of participants (MOs who attended the ECHO training), and key informant interviews (KIIs) with key stakeholders (MOs, hub-leaders, and trainers). These key informants were considered because they had involvement and experience with the ECHO training program for long COVID. Workpackage#2 (WP#2) assessed patient-side effect using a cross-sectional exploratory design wherein a quantitative survey and in-depth interviews (IDIs) were done with care-seekers for long COVID after (post) the training was conducted. Quantitative and qualitative data findings for assessing trained MOs, hub leaders, and trainers (Workpackage #1: Provider-side assessments) informed the first 1-to-5 levels of Moore’s framework. Patient satisfaction data from the exit interviews were for Level 6 of Moore’s framework (Table 1).

**Table 1:** Method of evaluation used in the study as per Levels of Moore’s Expanded Outcomes Framework for Assessing Learners.

Level of Evaluation	Indicators to be used	Method of Evaluation
Level 1: Participation	• Profile of healthcare providers trained	• Survey for participants’ profile • Interviews with the participants
Level 2: Satisfaction	• Participant satisfaction with training content • Degree to which expectations were met	
Level 3: Learning (knowledge)	• Improvement in knowledge levels (comparison of pre- and post-training scores)	• Survey: Pre and post-tests with participants • Interviews with the participants
Level 4: Competence	• Self-reported confidence to manage COVID-19 cases	
Level 5: Performance	• Self-reported change in practice due to knowledge gained	
Level 6: Patient health	• Patients’ satisfaction with treatment	• Survey: Exit Interviews with patients • In-depth interviews (IDIs) with patients

### Study settings

**WP#1:** Pre-post quantitative surveys were conducted across four states in India where the training was implemented, i.e., Madhya Pradesh (Central India), Gujarat (Western India), Karnataka (South-Western India), and West Bengal (Eastern India). Further, to understand the provider-side stakeholders’ perspectives on the effectiveness and impact of the training, key informant interviews (KIIs) were conducted at two sites: Gujarat (Ahmedabad Municipal Corporation; AMC) in Western India and West Bengal (Kolkata Municipal Corporation; KMC) in Eastern India.

**WP#2:** Health facilities at AMC and KMC were identified as study sites in consultation with local administrations and the ECHO India program team (Table in S5 Table).

### Study participants

**WP#1:** MOs enrolled for the training across the four states were requested to participate in the pre-training (baseline) quantitative survey. Those MOs who completed the pre-training (baseline) survey, consented to be contacted for the post-training (endline) survey, and attended at least three sessions of the ECHO long COVID training, were requested to complete the post-training quantitative survey. KIIs were conducted with MOs at public health facilities, trainers who conducted the ECHO long COVID training, and hub leaders who facilitated the training.

**WP#2:** Adult patients who had received long COVID treatment by the trained MOs were interviewed. These were patients who reported between January 2022 and July 2023, following an acute illness consistent with COVID-19 that continued beyond 3 weeks and provided consent to participate.

### Sample size and sampling technique

#### Quantitative study.

WP#1: Since long COVID-19 was a new condition, there were no studies available to suggest the knowledge, competence, and performance related to its management among MOs working in public health facilities in India. Hence, in WP#1 for determining the sample size of the MOs for the pre-post comparison, we assumed a 20-percentage point difference, i.e., from 50% at baseline to 70% at end line, with 90% power, 5% alpha (two-sided) and 0.8 correlation between baseline and end line measurements, and design effect of 1.5. A sample size of 201 each at baseline and end line (matched as pre-post) was calculated. Anticipating that data loss between baseline and end line could go up to 35% due to non-response and data cleaning, we decided to recruit about 270 participants at baseline so that we could expect to have data from at least 201 pre-post-matched participants for analysis.

The sample size formula is as follows:


$$I\frac{{\left(z_{\frac{\alpha }{\text{2}}}+z_{\beta }\right)}^{\text{2}}*(p_{\text{1}}\left(\text{1}-p_{\text{1}}\right)+(p_{\text{2}}\left(\text{1}-p_{\text{2}}\right)-\text{2}*r*\sqrt{p_{\text{1}}(\text{1}-p_{\text{1}})}*\sqrt{\begin{array}{c} p_{\text{2}}\left(\text{1}-p_{\text{2}}\right) \end{array}}}{{\left(p_{\text{1}}-p_{\text{2}}\right)}^{\text{2}}}*design\;effect$$

$p_{\text{1}}$: Baseline proportion

$p_{\text{2}}$: Endline proportion

$z_{\frac{\alpha }{\text{2}}}$: Z-score for the desired alpha level

$z_{\beta }$: Z-score for the desired power

r: Correlation between baseline and end line [16]

All the Medical Officers (MOs) in the first batch of training for each of the four states were requested to participate in WP#1.

**WP#2:** The sample size calculation was done assuming that at least 50% of the patients seeking out-patient care would have a favourable experience with the consultation for long COVID at the selected health facilities. With ±5% precision, 95% confidence level, and a design effect of 1.1, a sample size of 423 was calculated. Accounting for about 10% data loss/ non-response, we targeted to interact with 465 participants.

For sample calculation we used.


$$z^{\text{2}}*p*(\text{1}-p)/d^{\text{2}}\;$$

**z**: Represents the *z-score* associated with the desired confidence level; **p**: The estimated proportion of the population with the characteristic of interest and **d**: The *margin of error* or the acceptable difference between the sample proportion and the true population proportion [17].

A two-stage cluster sampling was used for collecting quantitative data. In the first stage, public health facilities from AMC and KMC were selected using simple random sampling, proportionate to the total number of facilities present in each of the two cities. Thus, in AMC, 15 of 26 facilities, and in KMC 30 of 75 facilities were selected. In the second stage for the selection of patients, consecutive sampling [18] was used with a target of 10 patients per facility on a randomly chosen day and time of the week.

#### Qualitative sample.

**WP#1:** The MOs, hub-leaders, and trainers for the KIIs were selected purposively with maximum variation among age, education, practice sites, and years of work experience. We tried to conduct as many KIIs as was operationally feasible during our visits.

**WP#2:** Purposive sampling was done to capture maximum variation among patients. At each study site, 10 patients were selected for the interviews – 5 men and 5 women –, 2 aged 18–44 years, 2 aged 45 years and above, and 1 of any age. This deliberate selection method ensured that a diverse range of patient experiences and perspectives were captured effectively.

### Intervention (The ECHO Training Package)

The intervention was a virtual, case-based learning program to train medical officers to identify, monitor, and manage long COVID syndrome among patients. A ‘hub and spoke’ structure was used wherein multidisciplinary teams of experts based at a regional academic medical center (the ‘hub’) used videoconferencing technology to engage with local healthcare providers (the ‘spokes’) (TeleECHO clinics) [19,20]. The curriculum was based on national and international guidelines for the clinical management of long COVID syndrome and developed in consultation with technical experts in COVID. It comprised six modules, i.e., managing general post-COVID sequelae, post-COVID gastrointestinal sequelae, post-COVID neurological sequelae, post-COVID cardiovascular sequelae, long-term effects of COVID on mental health, and co-morbid conditions. Knowledge was adapted as per local experience and transferred to community clinicians through two modalities in each session — short didactic presentations and group discussions of patient cases selected by the MOs from their clinical practice. The training presentation included text with visual learning methods, such as images, videos, and links to training resources. The training was implemented through a series of sessions in multiple batches across the four states. Each batch comprised about 80 participant MOs trained over five sessions spread over two months. A total of 860 MOs were, thus, trained through the program. The training was conducted in English. The trainer was chosen from each state and was fluent in the official language of the state (Madhya Pradesh-Hindi, Gujarat-Gujarati, Karnataka-Kannada, West Bengal-Bengali). The trainer used both English and the local language to communicate and address challenges faced by the participant MOs.

### Data collection

The study was conducted in compliance with the Helsinki Declaration and the Ethical Guidelines from the Indian Council of Medical Research, 2017, and approved by the institutional ethics committee of Jodhpur School of Public Health (JSPH) (ref: 10032/IRB/20–21, dated: 23rd August 2022).

**WP#1:** The MOs were requested to self-administer the questionnaire as ‘fill on their phones/ computers’ over the iECHO platform, before (pre-) (November 2022) and 1–3 months after (post-) training (March and April 2023) for each batch. The KIIs were conducted three months after the conclusion of the training (March and April 2023). Each KII was completed in about 45 minutes. The pre-post survey and KIIs were conducted in English.

**WP#2:** Field investigators (with social science degrees) with experience in conducting patient interviews were hired locally and were deployed at the selected health facilities in April and May 2023. All investigators were trained in a two-day face-to-face workshop for conducting patient interviews as part of the study. A hands-on exercise involving role-play scenarios was included for the investigators to practice their data collection techniques. On the days of data collection, the field investigators interviewed in the local language consecutive patients from outpatient departments after their consultation for long COVID with the identified MOs. The desired sample of quantitative exit interviews (n = 10) was completed first. The qualitative IDIs were done with subsequent patients. Thus, there were separate participants for the quantitative and qualitative components. The interviews were audio-recorded after consent was obtained from patients. Each interview lasted about 30 minutes. The data collectors took 15–20 days to complete data collection for WP #2.

### Study tools

#### Quantitative study tools.

**WP#1:** The pre-post quantitative survey tool was designed as a structured, closed-ended, electronic data collection form hosted online on the iECHO learning management platform. The tool included 66 questions on demographic characteristics (Level 1), satisfaction with the training (Level 2), knowledge (Level 3), skills/ competence (Level 4) and performance (Level 5), and 15 questions to assess technical knowledge (Level 3). The tool was developed by a review of literature followed by content validation by a panel of nine subject experts. The experts were requested to evaluate each item as ‘Very relevant’, ‘Quite relevant’, ‘Somewhat relevant’ and ‘Not relevant’. The Content Validity Ratio (CVR) of the whole instrument was calculated. Items with CVR > 0.7 were accepted and retained. Changes related to language, clarity, and relevance were made in the questionnaire based on the feedback from experts and pretesting with a group of MOs not included in the study. While knowledge was assessed as dichotomous response (Yes/ No), satisfaction, competence and performance were assessed with a 5-point Likert scale.

**WP#2:** The tool for the quantitative survey had two components, i.e., demographic characteristics and patient satisfaction questionnaire. The tool was adapted from other studies assessing patient satisfaction in public healthcare facilities [21,22]. The patient satisfaction questionnaire, further, had two components, i.e., technical quality and communication that were assessed using a 5-point Likert Scale. ‘Technical quality’ aimed to assess the beneficiary patient’s satisfaction with the consultation in terms of health examination, explanation of diagnostic tests, medication guidance, and information on side effects. ‘Communication’ aimed to evaluate satisfaction with the consultation in terms of the MO’s behavior, consultation time, doubt clarification, and explanation of follow-up care. It was designed as an electronic form on the Survey2Connect platform and was administered with due consent to the participants by trained field investigators using hand-held electronic devices (mobile phones) in Gujarati (AMC) and Bengali (KMC).

#### Qualitative study tool.

**WP#1:** Semi-structured guides were developed for the KIIs based on a review of the literature. The guide for MOs, trainers, and hub leaders delved into aspects such as their engagement, experience, practice changes, needs, and suggestions for improvement.

**WP#2:** The discussion guide for patients covered topics including post-COVID health issues, the experience of doctor visits, communication experiences, understanding of conditions, challenges faced, and suggestions for improvement. The tools were developed in English, translated to Gujarati and Bengali, pretested, and revised before finalization.

### Data Analysis

#### Quantitative data analysis.

Descriptive statistics were generated. Continuous data was summarized as mean and standard deviation and categorical data as frequency and proportion. Comparison of pre-post scores was done using paired t-tests for parametric continuous data with effect size, Wilcoxon’s signed rank test for non-parametric continuous data, and McNemar’s test for categorical data. The Likert responses were also compared as categorical variables using chi-squared tests by merging ‘Strongly Agree’ and ‘Agree’ as ‘Agree’, ‘Strongly Disagree’, and ‘Disagree’ as ‘Disagree’. A mixed effects linear regression model was applied to understand the factors influencing the MOs’ knowledge, competence, and performance. Mixed effects models include both fixed and random effects. The fixed effects are directly computed and are comparable to standard regression coefficients. The random effects are compiled based on their estimated variances and covariances. The state in which the MOs practiced (Madhya Pradesh, Gujarat, Karnataka, and West Bengal) was used as the group variable. The pre-training scores, gender, location of practice, and age were adjusted as independent variables. The difference was deemed to be statistically significant if p < 0.05. Statistical analysis was done using STATA Version 16 software (StataCorp. 2019. Stata Statistical Software: Release 16. College Station, TX: StataCorp LLC). The survey findings have been reported by following the STROBE checklist.

#### Qualitative data analysis.

The audio-taped qualitative data from the KIIs and IDIs was transcribed verbatim in the local language and then translated into English by professionals. These were anonymized and coded using Atlas.ti, version 8 software. Thematic analysis [23] was done using both deductive and inductive approaches. The analysis incorporated sociological theories of illness, support at the clinic, clinical relationships, access, and services. Two authors (SL and RM) familiarized themselves with the data and independently coded randomly selected transcripts using a set of apriori codes based on the discussion guide and study objectives. Using an inductive approach, initial codes were also generated, and emergent themes were identified and explored. Generated codes were refined as subthemes were identified and coalesced under appropriate themes. Final codes were organized in a thematic table. Patterns and connections within and between transcripts were explored and code saturation was agreed upon for the final analysis based on which the authors reviewed, defined, and finalized the themes, and identified illustrative quotes. The themes, including quotes (respondents’ exact words), represented the main findings. The researchers maintained a reflexive position throughout the analysis to minimize the risk of any presumptions that might affect the findings. The findings have been reported according to the Standards for Reporting Qualitative Research [24].

### Ethical Considerations

**Table 2:** Distribution of participants.

WP	Method		Madhya Pradesh	Gujarat	Karnataka	West Bengal	Total
**WP#1**	**Quantitative Survey** **(Trained Medical Officers)**	**Baseline**	123	72	25	51	271
		**Endline**	101	57	12	34	204
		**Follow-up Rate (%)**	82.1	79.2	48	66.7	**75.3**
**WP#1**	**Qualitative Interviews**	**Hub leaders**	–	1	1	1	3
		**Trainers**	1	2	1	3	7
		**Trained Medical Officers**	3	3	3	2	11
		**Total**	4	6	5	6	**21**
**WP#2**	**Quantitative survey**	**End line**	–	147	–	273	**420**
**WP#2**	**Qualitative Interviews**	**Patients**	–	10	–	10	**20**

*Patient-side assessments (WP#2):* A total of 420 beneficiary patients were surveyed from the two sites, i.e., AMC (n = 147) and KMC (n = 273). The IDIs were done with a total of 20 patients, i.e., 10 patients each from Kolkata (10 patients) and Ahmedabad (10 patients). The findings are presented according to Moore’s levels of evaluation (Table 2).

## Results

### Sample characteristics

*Provider-side effects (WP#1):* A total of 271 MOs participated in the pre- (baseline) assessment, with 204 completing the post- (end line) survey (follow-up rate: 75.3%). Consequently, the pre-post analyses for this assessment were conducted on 204 MOs. The overall mean age of the participants (n = 204) was 36.9 ± 8.6 years, with almost two-thirds (n = 150; 73%) being men. More than half (n = 120, 58.8%) were practicing at the Primary Health Centres (PHCs) followed by Community Health Centres (CHCs) (n = 56; 27.5%). Almost two-thirds (62.3%; n = 127) were posted in rural and 5.4% (n = 11) reported having been practicing in both rural and urban areas. Almost all the participants (n = 192; 94.1%) held Bachelor of Medicine, Bachelor of Surgery (MBBS) degrees with mean years of experience of 8.5 ± 7.1 years (Table in S1 Table). The KIIs were done with 11 MOs, 03 hub leaders and 07 trainers (Table 2).

### Level 1: Participation

The survey findings showed that some participants faced challenges while accessing the ECHO training, such as the day on which the sessions were held (n = 52; 25.5%), internet connectivity (n = 48; 23.5%), and the frequency of sessions (n = 41; 20.1%). The qualitative findings showed that participants were unable to focus when training sessions were spread across a longer duration of time. They also felt that hybrid sessions would be better where at least, one or two sessions could be conducted in person. (Table 3).

**Table 3:** Quotable quotes from medical officers trained in ECHO long COVID tele mentoring.

1: Participation	
**Active participation from MOs**	***“****The way it was done, having one of them as a presenter and involving them in the training.* ***There was active participation from the medical officers****. If you see the videos, the Q A session would be 30 minutes. That kind of participation in the first go is good to go I think.”* –Hub Leader, Karnataka.
**Challenges with duration of the training**	*“* ***The duration of the entire training should be less*** *. 1-2 months we may remember but later on, we might forget about it. However, the sessions are going on for a very long time, longer duration, about 7-8 months.” – Medical Officer, West Bengal.*
**Challenges with online training**	*“If the training is physical, I would have liked it more.* ***Because if it was physical training, the participation would be better. Whereas with video calling sessions whether we are present or not doesn’t matter much.*** *In case we have some other work in between the training, we have to attend. It happened with me 2-3 times the meeting was going on so I could not attend Echo training” – Medical Officer, Gujarat.*
**Challenges with the ECHO India platform**	“***ECHO India platform is not as easy as WhatsApp****. That is something… not that the platform is wrong or slow. Not platform issue but implementation issues.”–Hub Leader, Karnataka*
**Challenges with the timing of the training sessions**	*“It was weekly once. But the timing was decided virtually. We decided 12:30-1 would be ideal but after that, all the medical officers came with suggestions that they should have it between 2:30 and 3:30. So that the busy OPD gets over by 1:30-2 and then they have lunch. And then they start with this. We were a little calmer to listen and participate. That’s why they shifted to 1:30 and 2:30.” –Hub Leader, Karnataka.* *“* ***Time of the day was not suitable for me*** *. Echo had selected a good time as everyone would be at OPD in the morning. The time was in the afternoon. But at that time, we had other meetings as well.” – Medical Officer, Gujarat.*
**Level 2: Satisfaction**	
**Satisfaction with teaching method**	*“Excellent.* ***The teaching method is excellent and we can sit down and discuss how we can go ahead, improve ourselves further and set the bar higher****.”* –Trainer, West Bengal
**Trainers addressed queries**	*“Yes, definitely we had an opportunity for interaction. **The trainers were saying you can ask me questions/ doubts at any time**”* – Medical Officer, Gujarat
**Satisfaction with teaching content**	“***Curriculum was sufficient*** *as it covered most of the things.” – Medical Officer, Madhya Pradesh*
**Overall satisfaction**	“*Definitely they were happy at the end of every session.* ***2-3 of them messaged me personally saying the session was good.*** *They got to know something new.”* –Trainer, Gujarat
**Areas for improvement**	*“There also should be the feedback option where the trainees could share with us how this newly acquired knowledge has helped them in solving new cases better” –* Trainer, West Bengal.
**Level 4: Learning and Competence**	
***Improved knowledge and skill***	*“Yes, they were beneficial. When the patient comes who already has COVID and comes with post-COVID syndrome. Then we get to know****. We can diagnose the patient at the earliest and hence the patient will get cured at the earliest.”-*** *Medical Officer, Gujarat* *“Yes, definitely beneficial.* ***Because of the symptoms that we have seen in long COVID syndrome sessions, we could make out that this patient might have had long COVID syndrome****.” – Medical Officer, Madhya Pradesh* *“****The session has been beneficial to me. Not only professionally but personally too.*** *Because I had COVID. And people don’t know that they have been affected with COVID. We are alive. We got treated. But the body is left with sickness which we feel it’s due to daily things. If you have hair loss, period problems, also some spices taste are irritating you which you liked before COVID. So personally, it helped me that I could get to know its due to this.”* – Medical Officer, Madhya Pradesh
***Change in practice post-training***	*“****After these sessions, the issues have been recognized at the primary level itself*** *and there is increased use of anti-depression drugs at the OPD clinics which is an achievement.”* – Trainer, West Bengal
**Level 5: Performance**	
***Improved performance***	*“Earlier the patient would be scared after a COVID test thinking they would be sent to the hospital if the test result was positive. However****, after the training, we were better able to make them understand*** *that it’s not so and thus help them by lessening their fear.”- MO, Gujarat* *“There was one patient always complaining of body pain and nose irritation after recovering from COVID. We were treating him for allergies or for ENT-related issues. But he never got relief.* ***After attending the psychiatric part of the Long COVID training, we got to know why he was having the issues****. Though his problem still exists, now* ***we know the reason for his problem and we are able to counsel him better****.” – MO, Madhya Pradesh* *“The training is* ***relevant as it’s contextualized to our OPD setting****. If a patient has COVID, then the patient has mental stress. We have to counsel them even more.”-* Medical Officer, Gujarat
**Level 6: Patient satisfaction**	
***Technical quality of MOs***	*“The* ***doctor explained about medication, dosage, precautions, and treatment course in detail.*** *He also emphasized the importance of cleanliness and personal responsibility.”* – Male, Cough & Muscle Pain, AMC Patient *“I feel the treatment hasn’t helped because my health issue would have improved by now.” –Female, Fatigue and Breathing Problem, KMC patient* *“There is only one doctor who does all the treatment. Initially, I felt that she could cure me but* ***after so many days I am losing trust****.” –* Female, Breathing Problem, KMC Patient
***Communication of MOs***	*“The doctor was friendly. Despite my fears post-COVID,* ***he explained things so well, that it gave me a lot of mental support****.”* – Male, Diabetes, KMC Patient

### Level 2: Training Satisfaction

**Fig 1:**
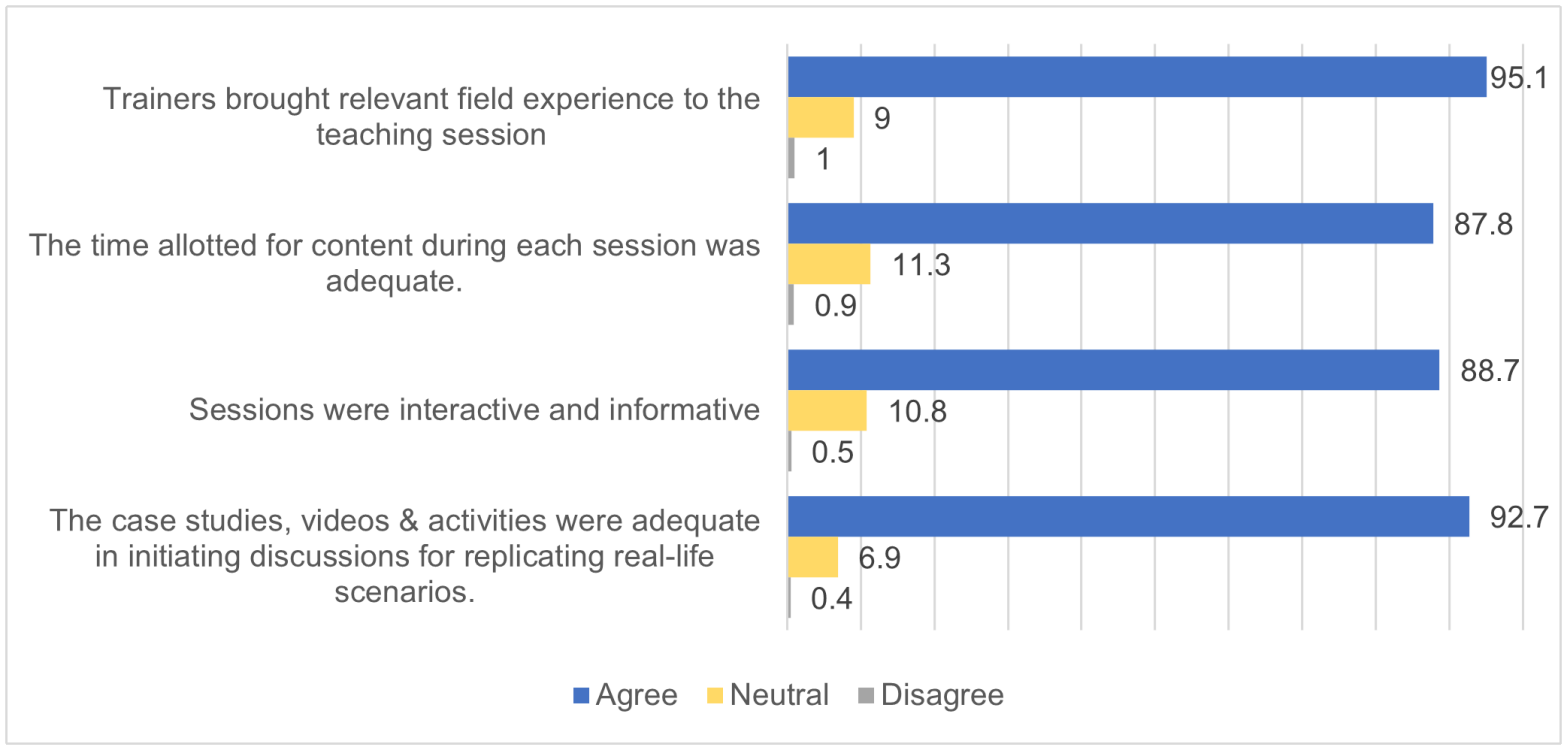
Satisfaction with the training content and environment of the ECHO long COVID tele-mentoring sessions as reported by the MOs.

The survey included four statements about the MOs’ satisfaction with the training content and environment. Almost all of the MOs agreed that the ECHO trainers brought relevant field experience to the teaching session, the time allotted for content during each session was adequate, the sessions held were interactive and informative, and the resources & activities were adequate in initiating discussions for replicating real-life clinical cases (Fig 1).

Of all the participants followed up, 93.1% (n = 190) informed that the training platform was accessible and 91.7% (n = 187) informed the training environment was conducive to learning, i.e., quiet, clear, and uninterrupted. While majority of the participants (96.6%, n = 171) were satisfied with the feedback mechanism, 8.8% (n = 18) reported that no feedback mechanism existed.

Qualitative findings resonated with most of the quantitative findings of WP#1 (Table 3). The key stakeholders reported that they found the ECHO training platform convenient and helpful. The MOs appreciated the interactive tele-mentoring sessions, which facilitated peer-to-peer learning and experience sharing. The model created opportunities for co-creation of long COVID knowledge network and professional support networks, and sharing clinical cases. The feedback mechanism encouraged transparency, allowing trainees to suggest training enhancements and communicate practical impacts on their professional activities.

### Level 3: Technical Knowledge and Skills

McNemar’s test was applied to understand the difference in the scores of technical knowledge and skills of participants between pre-ECHO and post-ECHO training. The test showed that there was a significant difference between the scores in 10 of the 15 questions under technical knowledge and skills (Table in S2 Table). When the overall scores of the 15 questions on knowledge and skills were taken together and compared, no significant difference (p = 0.807) between pre-ECHO and post-ECHO training scores was found.

### Level 4: Learning and Competence

There was a significant difference between the learning and competence scores of the participants between pre-ECHO and post-ECHO training in 07 of the 10 questions, when seen individually (Table in S3 Table). When the overall mean scores for the 10 questions of learning and competence were taken together for comparison, a significant difference (p < 0.05) between pre-ECHO scores and post-ECHO training (40.9 ± 3.1) scores was found.

The in-depth interviews with MOs also revealed that the training was beneficial as it helped them become more competent by improving their understanding of long COVID and thus enabling better diagnosis and management of patients with long COVID (Table 3).

### Level 5: Performance

**Fig 2:**
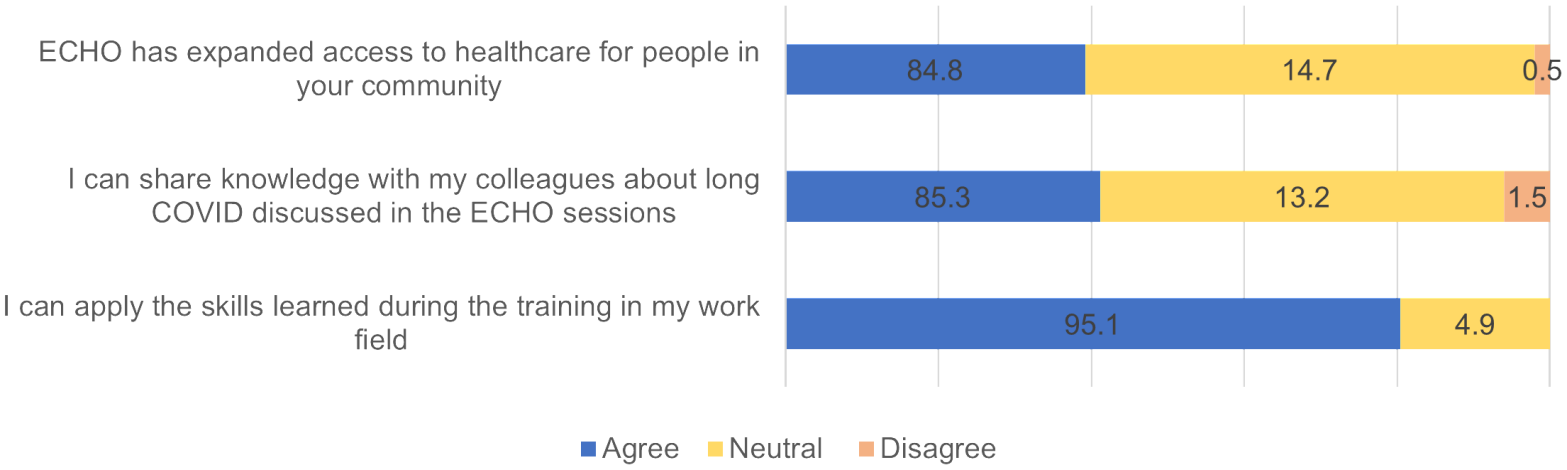
Self-assessment of the performance of the MOs after attending the ECHO long COVID tele-mentoring sessions.

Of the 204 participants, 75.5% (n = 154) MOs agreed that the ECHO training helped them in improving all the components of performance, i.e., they were able to apply the skills learned during the training in their work field, share the knowledge acquired in the ECHO sessions with other colleagues and also acknowledged that ECHO had expanded access to healthcare for people in the community. They also shared examples of instances when they felt better equipped to address the queries of the patients because of the training (Fig 2).

The test showed a significant difference between the pre-and post- training scores in 05 of the 07 questions on the participants’ attitude component when scored individually (Table in S4 Table). When the scores for all 7 questions were assessed, a significant difference (p < 0.05) in the self-reported attitude related to practice between pre-ECHO and post-ECHO training was found.

The mixed effects regression analysis found that the location of practice of the MOs (rural-reference category/ urban/ both) was the only significant predictor of the post-test technical knowledge (urban: β = 1.769; CI = 0.753, 2.786; p = 0.001) and performance scores (urban: β = 2.554; CI = 1.758, 3.350; p < 0.001) when adjusted for pre-test scores, gender, and age of the MOs. (Table in S6 Table).

### Level 6: Patient Satisfaction

**Fig 3:**
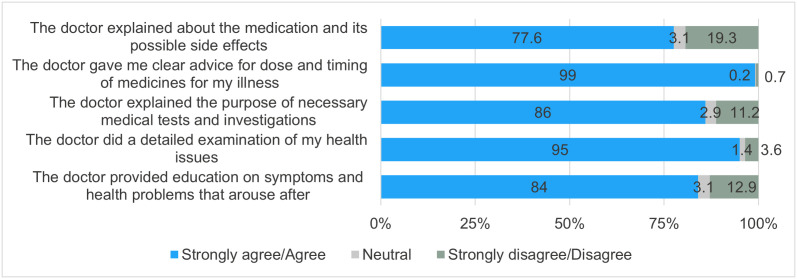
Technical quality question-wise responses as reported by the patients.

**Technical Quality:** Most of the patients reported positive experiences of their interaction with the MOs. Almost all of the participants (n = 416; 99%) agreed that the MOs provided clear instructions regarding the dosage and timing of medicines for their illness. Participants (n = 399; 95%) felt that the MOs conducted thorough examinations to assess their health issues. About two-thirds of the participants (n = 326; 77.6%) were satisfied with the MOs’ explanation regarding the prescribed medication and its potential side effects (Fig 3).

**Fig 4:**
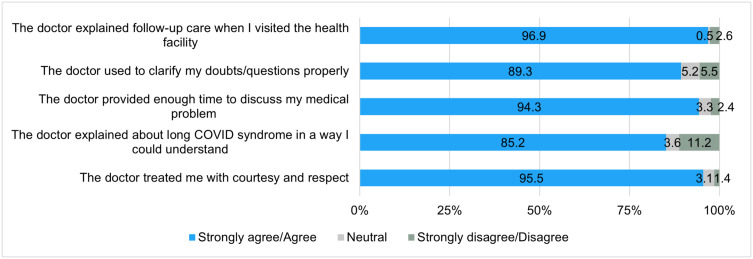
Communication satisfaction question-wise responses as reported by the patients. Patients appreciated that the doctors took the time to assess their health issues. They informed that the MOs assured them that essential treatments were free of cost.

**Communication:** The patients shared that the MOs had counselled them appropriately including detailed dietary advice that aligned with their lifestyle and religion. Almost all of them agreed that the doctors provided clear explanations regarding their follow-up care (n = 407; 96.9%), treated them with courtesy and respect (n = 401; 95.5%), and allocated sufficient time for the discussion of their medical concerns (n = 396; 94.3%). Most of the participants also agreed that doctors were able to clarify their doubts and questions (n = 375; 89.3%) and had explained the concept of long COVID syndrome in an understandable manner (n = 358; 85.2%) (Fig 4).

We compared patient satisfaction with the services received by the trained MOs between Kolkata and Ahmedabad to assess the effects of the ECHO CMS training in different health systems and local contexts. Patients from Kolkata had higher overall satisfaction levels with both the technical quality and communication skills of MOs. When stratified by gender, females demonstrated lower satisfaction levels than their male counterparts. Furthermore, patients who sought healthcare services on multiple occasions reported lesser satisfaction than those who sought consultation with a single visit. Patients who reported no incurred expenses displayed higher satisfaction levels compared to those who reported some expenditures. Significant differences (p < 0.05) were found across these parameters (Table 4).

**Table 4:** Comparison of satisfaction mean scores according to patient profile.

		Mean Score			Mean Score					
		Technical Quality	Mean diff	P-value*	Communication	Mean diff	P-value*	Total	Mean diff	P-value*
City	Kolkata (n = 213)	10.5 (4.0)	2.8	<0.001	9.3 (3.5)	1.7	<0.001	19.9 (7.1)	4.6	<0.001
	Ahmedabad (n = 207)	7.7 (2.3)			7.6 (2.3)			15.3 (4.5)		
	Male (n = 163)	9.8 (3.7)	1.1	0.002	9.2 (3.5)	1.1	<0.001	19.0 (6.9)	2.2	<0.001
	Female (n = 257)	8.7 (3.4)			8.1 (2.8)			16.8 (5.9)		
Number of times sought care	Once (n = 95)	10.0 (3.3)	1.1	0.006	9.5 (3.1)	1.3	<0.001	19.5 (6.0)	2.4	0.001
	More than once (n = 325)	8.9 (3.6)			8.2 (3.1)			17.1 (6.4)		
Amount spent	Nothing spent (n = 299)	10.2 (3.7)	1.5	0.002	9.2 (3.1)	1.0	0.004	19.4 (6.4)	2.5	<0.001
	Spent something (n = 121)	8.7 (3.5)			8.2 (3.1)			16.9 (6.2)		

*P-value was calculated using a t-test.

All p values are significant (p < 0.05).

## Discussion

The novelty of the long COVID syndrome has resulted in limited knowledge and understanding of the management of patients with long COVID [25]. The study findings revealed a significant increase in the scores of knowledge, learning and competence, performance, and attitude of the MOs towards assessment and management of long COVID between pre- and post-ECHO training across all states. This indicated a positive impact of the training and is in alignment with the findings of previous ECHO studies [26–28]. The results indicated that while several individual knowledge items improved significantly post-training, the composite knowledge score did not show a statistically significant change. This could be due to the high baseline knowledge (ceiling effect) in some items. While some individual knowledge items showed significant improvements, others, such as knowledge of the side effects of medications, did not improve, resulting in a non-significant change in the composite score. In our study, the MOs practicing in urban areas scored higher in technical knowledge and performance, compared to their rural counterparts. The challenges, such as poor internet connectivity, frequent power outages, limited technological infrastructure and digital literacy, hamper digital learning initiatives for health professionals in rural areas [29,30]. The inclusion of targeted support in the form of familiarising rural participants with the digital platforms, recorded sessions, and post-training engagement for problem-solving can be focused on in future training programs.

The stakeholder feedback was overall positive as reported in other evaluations of the ECHO program [31]. A few participants and trainers felt that the feedback mechanism included in the training was not sufficient. The feedback mechanism could be further strengthened by reporting the clinical application of the knowledge acquired through the training. Based on the inputs. optimizing the tele-mentoring platform for better interactivity, expanding program resources, and addressing logistical barriers seem to be the way forward for program refinement as has been reported in other ECHO evaluations [32]. Other studies have reported that offering ECHO training for both synchronous and asynchronous viewing can decrease logistical and participation barriers [33].

The evaluation also indicated a substantial level of satisfaction among providers participating in the ECHO tele-mentoring program. This has also been reported in other evaluations of ECHO COVID programs [26,34–36]. These findings can inform healthcare policies related to continuing medical education and capacity building, potentially leading to broader adoption of similar tele-mentoring initiatives to address other healthcare challenges [37–40].

The MOs reported an improvement in their attitude and ability to manage long COVID cases. The assessments of the work package 2 showed that the patients were satisfied with the technical quality and communication skills of the trained MOs, and this was in alignment with other studies [41–44]. The qualitative data showed that after training, the MOs focused on counselling using empathy (learned from the training) for treating long COVID. The patients also described how alleviating mental stress through proper communication with the MO contributed to a positive experience of receiving care for long COVID. A scoping review reported positive patient outcomes in studies that used interventions to support mental health among people with long COVID [45]. Overall, the findings from providers and patients suggested that the training improved the patient-centricity of services for long COVID [46].

The ECHO tele-mentoring initiative can serve as a model for capacity building in public health facilities in low-middle-income countries and has the potential to rapidly scale up capacity building in both long COVID syndrome and other healthcare challenges [47–51]. The lessons learned from this evaluation can guide future program improvements and inspire the implementation of similar initiatives in the field of public health and medical education [52,53].

### Strengths and limitations

The quasi-experimental design, involved a pre- and post-training assessment, without a control group. The overall follow-up rate for MOs was 75%, with variations across states, and 100% follow-up could not be achieved. In some intervention sites such as West Bengal (66.7%) and Karnataka (48.0%), the follow-up rates were lower. Previous research on the ECHO model has usually focussed on participant-level outcomes, with less attention given to patient-level perspectives. This study explored patient satisfaction within the context of the program and the benefits of the ECHO tele-mentoring model on improvements in the delivery of healthcare at public facilities. However, for patient-level data, a one-time cross-sectional assessment was conducted without comparing pre- and post-training scores or having a control group.

## Conclusion

The ECHO Long-COVID tele-mentoring of MOs in primary care had led to improvements in technical knowledge and skills, learning and competence, performance, and satisfaction of participants. The study also concluded that patients who received care from these trained MOs were satisfied with the consultations they received for long COVID. The ECHO model has the potential to serve as a reference model for training scale-up for relatively novel conditions like long COVID within the public health system in LMICs like India.

## Supporting information

S1 Table Profile of the Participant Medical Officers. (DOCX)

S2 Table Questions on technical knowledge and skills. (DOCX)

S3 Table Knowledge and self-efficacy after ECHO-training on Long COVID. (DOCX)

S4 Table Self-reported attitude related to work practice after participation in the ECHO training. (DOCX)

S5 Table Distribution of health facilities in Ahmedabad (AMC) and Kolkata (KMC). (DOCX)

S6 Table Mixed effects linear regression model of measures at pre and post-test from the ECHO training intervention. (DOCX)
