# Supplementary material for: Effects of the ECHO tele-mentoring program on Long COVID management in health facilities in India: A mixed-methods evaluation
Source: PLoS One. 2025 Nov 11;20(11):e0331293. doi: 10.1371/journal.pone.0331293 (PMC12604793; doi:10.1371/journal.pone.0331293)
Supplement: S1 Table — (DOCX) [file pone.0331293.s001.docx]

S1 Table. Profile of the Participant Medical Officers

| Characteristics |  | n (%) |
| --- | --- | --- |
| Age, mean (SD) |  | 36.9 (8.6) |
| Gender | Male | 150 (73.5%) |
|  | Female | 54 (26.5%) |
| Site of Practice | SC | 7 (3.4%) |
|  | PHC | 120 (58.8%) |
|  | CHC | 56 (27.5%) |
|  | DH/SDH | 14 (6.9%) |
|  | Other specify | 07 (3.4%) |
| Location of practice | Rural | 127 (62.3%) |
|  | Urban | 66 (32.4%) |
|  | Both | 11 (5.4%) |
| Education Qualification | MBBS | 192 (94.1%) |
|  | MD | 11 (5.4%) |
|  | Other specify | 01 (0.5%) |
