# Supplementary material for: Effects of the ECHO tele-mentoring program on Long COVID management in health facilities in India: A mixed-methods evaluation
Source: PLoS One. 2025 Nov 11;20(11):e0331293. doi: 10.1371/journal.pone.0331293 (PMC12604793; doi:10.1371/journal.pone.0331293)
Supplement: S2 Table — (DOCX) [file pone.0331293.s002.docx]

S2 Table**.** Questions on technical knowledge and skills

| Sr no | Questions | Pre-ECHO  *n (%) | Post-ECHO  *n (%) | **P-value |
| --- | --- | --- | --- | --- |
| 1 | Long-COVID syndromes are signs and symptoms of COVID 19 infection that continues for more than 12 weeks | 189 (92.6%) | 174 (85.3%) | <0.05 |
| 2 | A 35-year-old patient had COVID-19 and experienced 2 weeks of fever, sore throat, coughing, and shortness of breath while she had isolated herself in basement. After the fever, she alternated between feeling better at times and having extreme fatigue. Some of the symptoms become worse after she exercises. What should her doctor do? | 48 (23.5%) | 73 (35.8%) | <0.05 |
| 3 | Who amongst the following is most likely to develop Long COVID? | 166 (81.4%) | 140 (68.6%) | <0.05 |
| 4 | Which of the following is a pulmonary complication that can be associated with Long Covid? | 152 (74.5%) | 160 (78.4%) | 0.358 |
| 5 | How to manage patients with the complaint of dyspnoea after recovering from COVID-19? | 121 (59.3%) | 152 (74.5%) | <0.05 |
| 6 | A 45-year-old male patient had a mild case of COVID-19, with no fever or loss of smell or taste but exhaustion and a severe cough. The intense coughing spells and bronchospasms continued after he recovered from COVID-19. What should be the management of this patient? | 139 (68.1%) | 124 (60.8%) | 0.1078 |
| 7 | The cardinal symptoms of post-acute COVID-19 respiratory sequelae include the following | 158 (77.5%) | 150 (73.5%) | 0.339 |
| 8 | Which of the following is nephrological complications associated with long COVID | 89 (43.6%) | 137 (67.2%) | <0.05 |
| 9 | A patient coming with a history of diabetes and symptoms of fatigue, joint and muscle pain after recovering from COVID-19. How do you manage this condition? | 101 (49.5%) | 104 (51.0%) | 0.755 |
| 10 | Which of the following is commonly reported as a neurological symptom post COVID-19 | 138 (67.6%) | 158 (77.5%) | <0.05 |
| 11 | A patient was tested positive for COVID-19 infection and was given azithromycin along with other drugs. Which of the following can be the common side effects of the drug? | 136 (66.7%) | 115 (56.4%) | <0.05 |
| 12 | Can liver injury due to COVID-19, drugs or alternative medication intake be a reason for GI related post COVID manifestations? | 171 (83.8%) | 116 (56.9%) | <0.05 |
| 13 | Delayed reaction to a stressful event can be associated with | 105 (51.5%) | 129 (63.2%) | <0.05 |
| 14 | Meena is 30 years old and was fine until six months ago when she was diagnosed with COVID-19 and hospitalized. Then she feels tired all the time and has lost interest in life. She cannot sleep, and she has lost the taste for food, which she used to love. She has also lost interest in cooking because she can’t concentrate. Sometimes she feels like taking her life. What would she be | 123 (60.3%) | 77 (37.7%) | <0.05 |
| 15 | Which of the following must be done to cope with post COVID-19 mental health conditions | 155 (76.0%) | 167 (81.9%) | 0.163 |

*n- frequency

**P-value has been calculated using McNemar’s Test
