# Supplementary material for: Effects of the ECHO tele-mentoring program on Long COVID management in health facilities in India: A mixed-methods evaluation
Source: PLoS One. 2025 Nov 11;20(11):e0331293. doi: 10.1371/journal.pone.0331293 (PMC12604793; doi:10.1371/journal.pone.0331293)
Supplement: S3 Table — (DOCX) [file pone.0331293.s003.docx]

S3 Table. Knowledge and self-efficacy after ECHO-training on Long COVID

| **SNo.** | **Questions** | **Pre-ECHO** | **Post-ECHO** | **P-value*** |
| --- | --- | --- | --- | --- |
| 1 | I feel confident in understanding cardiovascular, pulmonary, neurological, and mental health complications associated with long COVID conditions. | 3.82 ± 0.7 | 4.05 ± 0.4 | < 0.05 |
| 2 | I feel confident in understanding the common side-effects of COVID-19 drugs causing long COVID syndrome. | 3.86 ± 0.7 | 4.14 ± 0.6 | < 0.05 |
| 3 | I feel confident in recognizing signs and symptoms of long COVID syndrome. | 4.02 ± 0.6 | 4.17 ± 0.6 | < 0.05 |
| 4 | I feel confident in doing a clinical assessment of long COVID syndrome | 3.92 ± 0.7 | 4.12 ± 0.6 | < 0.05 |
| 5 | I feel confident in identifying tests and investigations required for the assessment of long COVID syndrome | 3.87 ± 0.7 | 4.00 ± 0.6 | 0.0659 |
| 6 | I feel confident in prescribing medicines and treatment for patients with long COVID syndrome | 3.80 ± 0.8 | 4.08 ± 0.6 | < 0.05 |
| 7 | I feel confident in giving advice and managing patients with long COVID syndrome | 4.02 ± 0.6 | 4.11 ± 0.5 | 0.1174 |
| 8 | I feel confident in assessing treatment response in patients with long COVID syndrome | 3.86 ± 0.7 | 4.08 ± 0.5 | < 0.05 |
| 9 | I feel confident that I am using best-practice approaches for the management of long COVID syndrome | 3.90 ± 0.7 | 4.02 ± 0.6 | < 0.05 |
| 10 | I feel confident in my ability to educate the patients and clarify their questions about long COVID syndrome | 4.10 ± 0.6 | 4.09 ± 0.5 | 0.8643 |
|  | **Over All (Total)** | **39.15 ± 4.5** | **40.89 ± 3.1** | < 0.05 |

*P-value has been calculated using paired t-test
