# Supplementary material for: Effects of the ECHO tele-mentoring program on Long COVID management in health facilities in India: A mixed-methods evaluation
Source: PLoS One. 2025 Nov 11;20(11):e0331293. doi: 10.1371/journal.pone.0331293 (PMC12604793; doi:10.1371/journal.pone.0331293)
Supplement: S4 Table — (DOCX) [file pone.0331293.s004.docx]

S4 Table. Self-reported attitude related to work practice after participation in the ECHO training

| **Sl No.** | **Questions** | **Pre-ECHO** | **Post-ECHO** | **P-value*** |
| --- | --- | --- | --- | --- |
| 1 | Symptom management approaches can improve long COVID conditions. | 4.21 ± 0.63 | 4.36 ± 0.57 | < 0.05 |
| 2 | Rehabilitation plan including physical therapy and exercises should be indicated to patients. | 4.16 ± 0.65 | 4.03 ± 0.67 | < 0.05 |
| 3 | Oxygen therapy is delivered when exertional oxygen saturation is below 90% | 3.95± 0.80 | 4.02± 0.04 | 0.2887 |
| 4 | Vaccination should not be taken even after 3 months post COVID-19 recovery | 3.10 ± 1.25 | 3.64 ± 1.12 | < 0.05 |
| 5 | Steroids can be administered in diabetes patients for a longer duration of time. | 2.95 ± 1.12 | 3.48 ± 1.09 | < 0.05 |
| 6 | FAST is an important method in stroke management | 3.80 ± 0.70 | 3.94 ± 0.56 | < 0.05 |
| 7 | Counselling on lifestyle components such as nutrition, sleep, and stress reduction is advisable. | 4.05± 0.76 | 4.09± 0.61 | 0.565 |
|  | **Overall** | **26.22 ± 2.64** | **27.55 ± 2.57** | < 0.05 |

*P-value has been calculated using paired t-test
