# Supplementary material for: Effects of the ECHO tele-mentoring program on Long COVID management in health facilities in India: A mixed-methods evaluation
Source: PLoS One. 2025 Nov 11;20(11):e0331293. doi: 10.1371/journal.pone.0331293 (PMC12604793; doi:10.1371/journal.pone.0331293)
Supplement: S5 Table — (DOCX) [file pone.0331293.s005.docx]

S5 Table. Distribution of health facilities in Ahmedabad (AMC) and Kolkata (KMC)

| City | Facility | Total centres | No. Of centres selected | Sample | Total |
| --- | --- | --- | --- | --- | --- |
| Ahmedabad | *UPHC | 17 | 15 | 147 | **420** |
|  | **CHC | 9 |  |  |  |
| Kolkata | UPHC | 74 | 30 | 273 |  |
|  | ***KMC HQ | 01 |  |  |  |

*UPHC- Urban Public Health Facility

**CHC- Community Health Centre,

***KMC HQ- Kolkata Municipal Corporation HeadQuarter
