# Supplementary material for: Effects of the ECHO tele-mentoring program on Long COVID management in health facilities in India: A mixed-methods evaluation
Source: PLoS One. 2025 Nov 11;20(11):e0331293. doi: 10.1371/journal.pone.0331293 (PMC12604793; doi:10.1371/journal.pone.0331293)
Supplement: S6 Table — (DOCX) [file pone.0331293.s006.docx]

S6 Table. Mixed effects linear regression model of measures at pre and post-test from the ECHO training intervention

|  | **Technical knowledge** | | **Learning & competence** | | **Attitude & performance** | |
| --- | --- | --- | --- | --- | --- | --- |
|  | ᵦ (95% CI) | *p* value | ᵦ (95% CI) | *p*  value | ᵦ (95% CI) | *p* value |
| Pre-scores | 0.920 (-.040,.224) | 0.174 | .083 (0.009,0.176) | 0.079 | 0.019 (-.106,.144) | 0.765 |
|  |  |  |  |  |  |  |
| Gender | 0.251 (-.748,1.250) | 0.622 | -.429 (-1.463,0.604) | 0.415 | -.636 (-1.398,.126) | 0.102 |
|  |  |  |  |  |  |  |
| Location of practice |  |  |  |  |  |  |
| Urban | 1.769 (.753,2.786) | **0.001** | 0.414 (-1.070,1.898) | 0.584 | 2.554 (1.758,3.350) | **<0.001** |
| Rural and urban | -.670 (-2.856,1.514) | 0.547 | 0.189 (-1.967,2.346) | 0.863 | 0.111 (-1.560,1.784) | 0.896 |
|  |  |  |  |  |  |  |
| Age | -.006 (-.055,.042) | 0.806 | 0.002 (-.046,0.050) | 0.927 | -.018 (-.056,.018) | 0.329 |
